# Book Reviews

**Published:** 1918-02

**Authors:** 


**Medical Directory of N. Y., N. J. and Conn.** Published by the Medical Society of the State of N. Y., revised to Oct. 24, 1917.

This well known directory is one of the most important activities of the State Society. Its accuracy is diminished this year by the considerable number of men in military service. This is inevitable, but we trust that by next fall, it will be feasible to include a military directory. Our readers are requested to report changes due to death, removal, false listing, etc., and to call the attention of men with medical degrees who are not in practice, to the special list provided to meet such cases. There are 14,421 physicians in N. Y. state, 7,968 in Greater N. Y. City, 6,453 in the rest of the state. The difference is greater than in the past. 2,792 physicians are listed for N. J. and 1,442 for Conn.

---

**Safe-Guarding the Special Senses.** Henry O. Reik, M. D., Baltimore (now Captain M. R. C.). Published by the F. A. Davis Co., 1912. 123 pages, illustrated.

As we have reviewed books 200 years old, we make no apology for referring to one off the press for several years. Military developments have made this subject one of particular importance, not only for the army itself but in the interests of the potential militia and the great army of industry. While not a technical work, it is far from being a mere lay educator, and general indications for medical and surgical treatment are included. The work would be classified as one of specialized hygiene, but is not arbitrarily limited in this direction, and it serves a purpose not met, so far as we are aware, by any other book, in quite so satisfactory a way.

**State Department of Health** of New York, 36th Annual Report, Vols. 1, 2, 3, for the year of 1915.

